# In-situ, time resolved monitoring of uranium in BFS:OPC grout. Part 2: Corrosion in water

**DOI:** 10.1038/s41598-018-27636-2

**Published:** 2018-06-18

**Authors:** C. A. Stitt, C. Paraskevoulakos, A. Banos, N. J. Harker, K. R. Hallam, H. Pullin, A. Davenport, S. Street, T. B. Scott

**Affiliations:** 1 Interface Analysis Centre, H. H. Wills Physics Laboratory, University of Bristol, Bristol, UK; 2 European Synchrotron Radiation Facility, Grenoble, Rhône-Alpes France; 3 School of Metallurgy and Materials, University of Birmingham, Edgbaston, Birmingham, UK

## Abstract

To reflect potential conditions in a geological disposal facility, uranium was encapsulated in grout and submersed in de-ionised water for time periods between 2–47 weeks. Synchrotron X-ray Powder Diffraction and X-ray Tomography were used to identify the dominant corrosion products and measure their dimensions. Uranium dioxide was observed as the dominant corrosion product and time dependent thickness measurements were used to calculate oxidation rates. The effectiveness of physical and chemical grout properties to uranium corrosion and mobilisation is discussed and Inductively Coupled Plasma Mass Spectrometry was used to measure ^238^U_(aq)_ content in the residual water of several samples.

## Introduction

Uranium metal is a significant contributor to Intermediate Level nuclear Waste (ILW) at Sellafield, UK. Along with other reactive metals such as Magnox cladding and aluminium, the uranium is encapsulated in a Blast Furnace Slag (BFS) and Ordinary Portland Cement (OPC) mixed grout and stored in stainless steel containers above ground, awaiting disposal via the potential development of a Geological Disposal Facility (GDF)^1^. In a prequel to this study^2^, we investigated the corrosion behaviour of uranium encapsulated in grout and exposed to water vapour, reflecting conditions during surface storage of the waste. We refer the reader to this paper for further background information and sample history. From the results of this paper, we concluded that under water vapour conditions uranium metal corrodes via the anoxic oxidation reaction displayed in Equation 1. In agreement with other authors^3–5^, we also found the ingress of oxidising species from the water vapour environment was limited by the reducing permeability of the progressively hydrating grout. As a result, the uranium oxidation rate was observed as initially rapid, but decreased over time, and near 50 weeks plateaued at a rate believed to reflect the steady state exchange of gases through the near-matured grout.

1 $$U+2{H}_{2}O\to U{O}_{2}+2{H}_{2}$$

These findings are important because Equation 1 yields hydrogen which can potentially become trapped in the grout pores and furthermore, if concentrations are allowed to increase over long periods of time, uranium hydride (UH_3_) may form. This compound is a black powder which has been known to spontaneously ignite in oxidising conditions^6,7^ and, in addition to hydrogen gas, significantly increases the risk for future transportation, storage and disposal of the ILW containers.

The UK’s current plan is to build a GDF for the disposal of nuclear waste. To ensure the safe containment of such material, the change in environmental conditions must be considered in predictive risk modelling. On completion and after loading the waste, the GDF vault will most likely be backfilled with an OPC based material, thus preventing further reaction with atmospheric gases^8^. Temperatures are expected to rise to 80 °C as a result of curing cementitious materials and in time, anoxic and chlorinated groundwaters are expected to ingress, progressively saturating the entire facility^3^. Although now buried underground, the risks here are potentially forming UH_3_ stores (which could later be accidently discovered), and aqueous release of uranium into the surrounding environment if the steel containment fails.

The encasing grout of an ILW package performs as both a chemical and physical barrier against uranium release into the surrounding environment. It is therefore important to understand the potential mechanisms of uranium immobilisation and mobilisation within the grout matrix and ultimately the factors which affect them. Immobilisation of uranium in grout can occur through precipitation of a salt, solid solution (adsorption and incorporation of uranium into grout hydration phases) and simple sorption to hydrated grout surfaces through electrostatic surface complexation or ion exchange^9,10^. However, uranium mobilisation can be mediated through complexation with strongly oxidising ligands in the aqueous phase (e.g. carbonates^11,12^, sulphates^12^ and phosphates^12^) or kept within solution by weaker complexation ligands (e.g. hydroxyl ions^13^)^9,10,14^. Singularly, these processes are complex and dependent on a multitude of environmental conditions, each of which must be investigated.

Pourbaix diagrams provide a good indication of the thermodynamically stable species of uranium under various aqueous pH and Eh conditions and according to the U-H_2_O diagram presented by Sutton^15^, under high pH and low Eh conditions uranium corrosion tends towards oxidation to UO_2_. However, the effect of grout on the U-H_2_O system and furthermore the dominant corrosion products of uranium and their rate of formation in these conditions are not well known. In the following experiment we address this problem by performing an *in-situ* study of uranium corrosion in grout under full water submersion conditions, in an attempt to reflect the main features of a saturated GDF. Synchrotron x-ray powder diffraction (XRPD) and tomography (XRT) were used to identify and analyse the morphology and location of the dominant uranium corrosion products over a 47 week time period. We then used Inductively Coupled Plasma Mass Spectrometry (ICP-MS) to measure the concentration of aqueous uranium species in residual waters.

## Results

A series of uranium rods (0.5 mm × 0.5 mm × 20 mm in dimension) were encapsulated in an BFS:OPC grout, then fully submersed in de-ionised water for 2 to 47 weeks. Each sample was analysed on the I12, Joint Engineering, Environment and Processing (JEEP) beam line, at the Diamond Light Source (DLS) over two separate beam times. In total 7 samples were analysed, which were split into two groups as shown in Table 1.

**Table 1:** A summary of the 7 uranium metal samples, including the sample origin, type of metal, water vapour exposure length and the beam time examined.

Sample name	Pre-treatment	Exposure length (weeks)	Beam time examined
A3L	As-received	3	1
A6L		6	1
A47L*		47	2
N2L	Nitric acid etched	2	2
N6L		6	2
N12L		12	2
N22L		22	2

*Sample A3L was re-analysed on the second beam time after further water submersion. This was renamed to A47L.

Before encapsulation in grout, three samples retained an as-received corrosion layer on the metal surface thereby reflecting uranium fuel which is pre-corroded before waste packaging (Group A in Table 1). Four more samples represented recently de-canned uranium fuel, and were pre-treated with nitric acid prior to grout encapsulation (Group N in Table 1). The names of the two sample groups begin with a letter A and N respectively followed by a number which accounts for the number of weeks exposed to water vapour.

**Figure 1:**
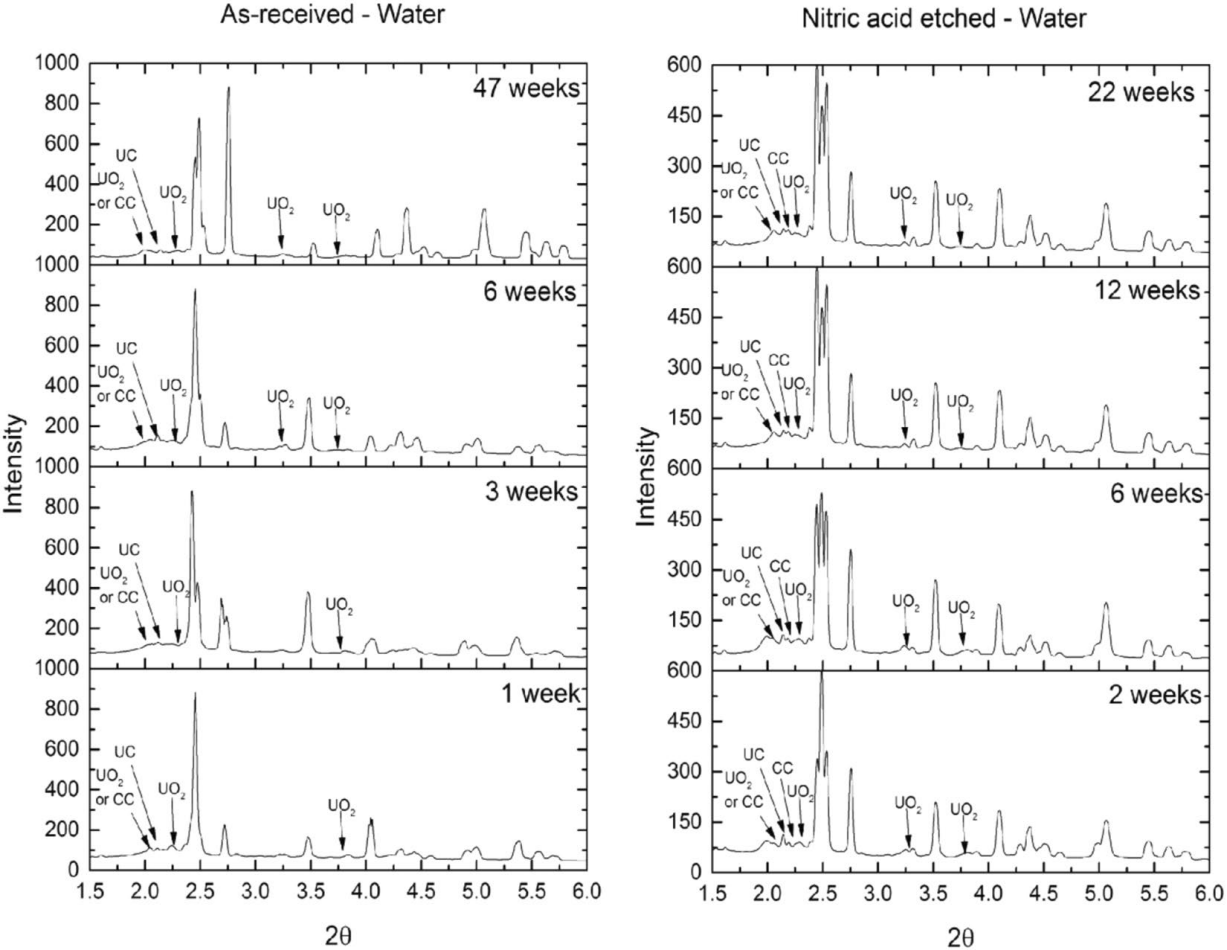
XRPD exhibiting the evolution of as-received and nitric acid etched, grout encapsulated uranium when submersed to de-ionised water over time. All unlabelled peaks are attributed to uranium metal. CC = Calcium carbonate (CaCO_3_).

XRPD line scan data across each uranium rod and grout were averaged for each sample and shown in Fig. 1. Over the 47-week period, UO_2_ was identified as the dominant corrosion product and no strong evidence of UH_3_ was observed.

**Figure 2:**
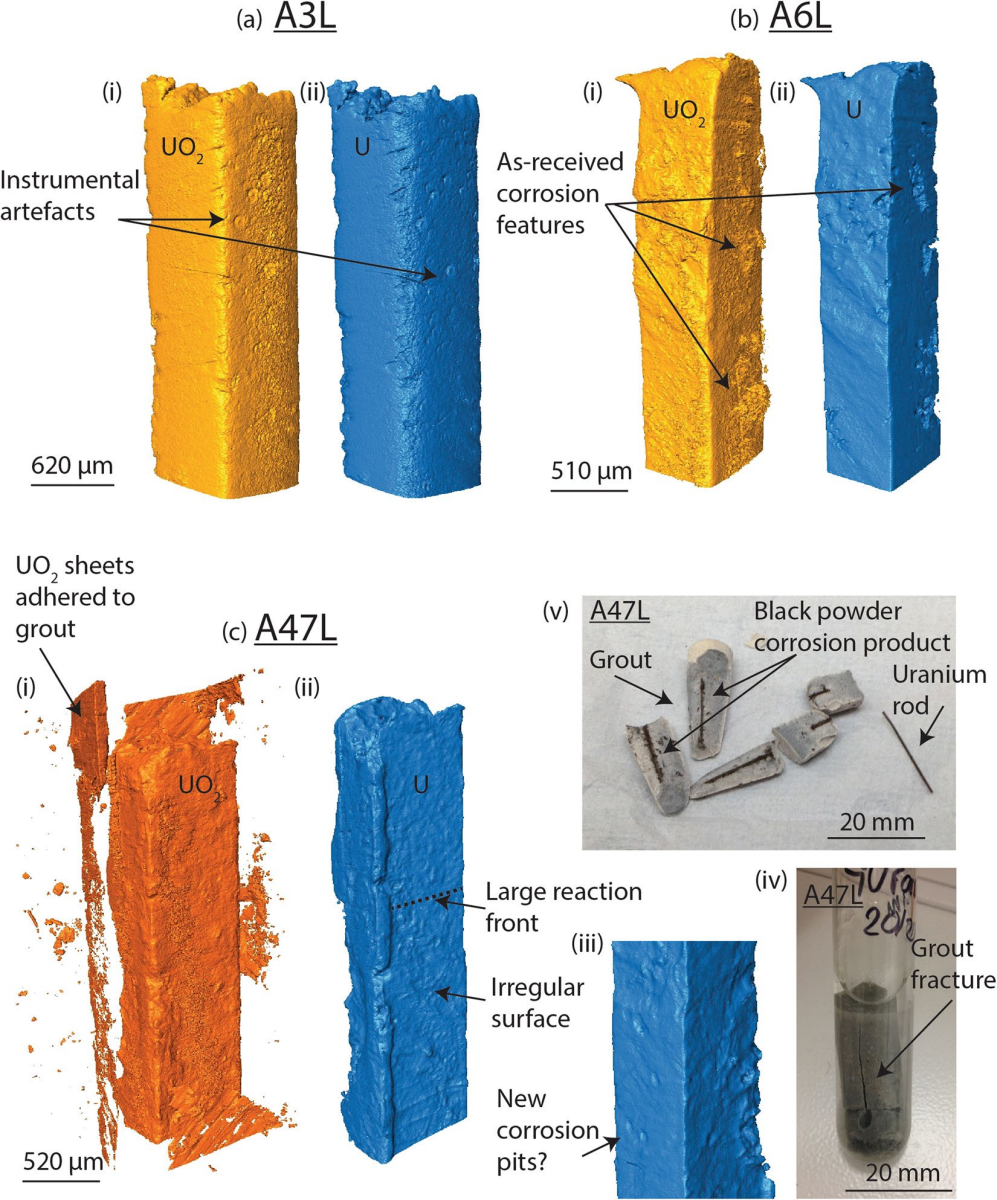
3D renders of the as-received uranium encapsulated in grout, after submersion in de-ionised water for time periods up to 47 weeks. Each sample is displayed in pairs; yellow or orange representing the corrosion product (UO_2_) and blue the uranium metal. Since the XRT quality was significantly lower using higher beam energies (115.6 keV), the corrosion product renders from the first beam time are highlighted in yellow ((**a**)(i) and (**b**)(i)), whilst the sample examined using lower energy (113.3 keV – beam time 2) is displayed in orange ((**c**)(i)). In general, a decrease in the number of instrumental artefacts, sharper imaging and reduced x-ray absorption by the uranium metal was observed using lower energies, allowing clearer identification and analysis of the corrosion product morphology. Photographs (**c**)(iv-v) show sample A47L before and after removal from the reaction cell respectively.

**Figure 3:**
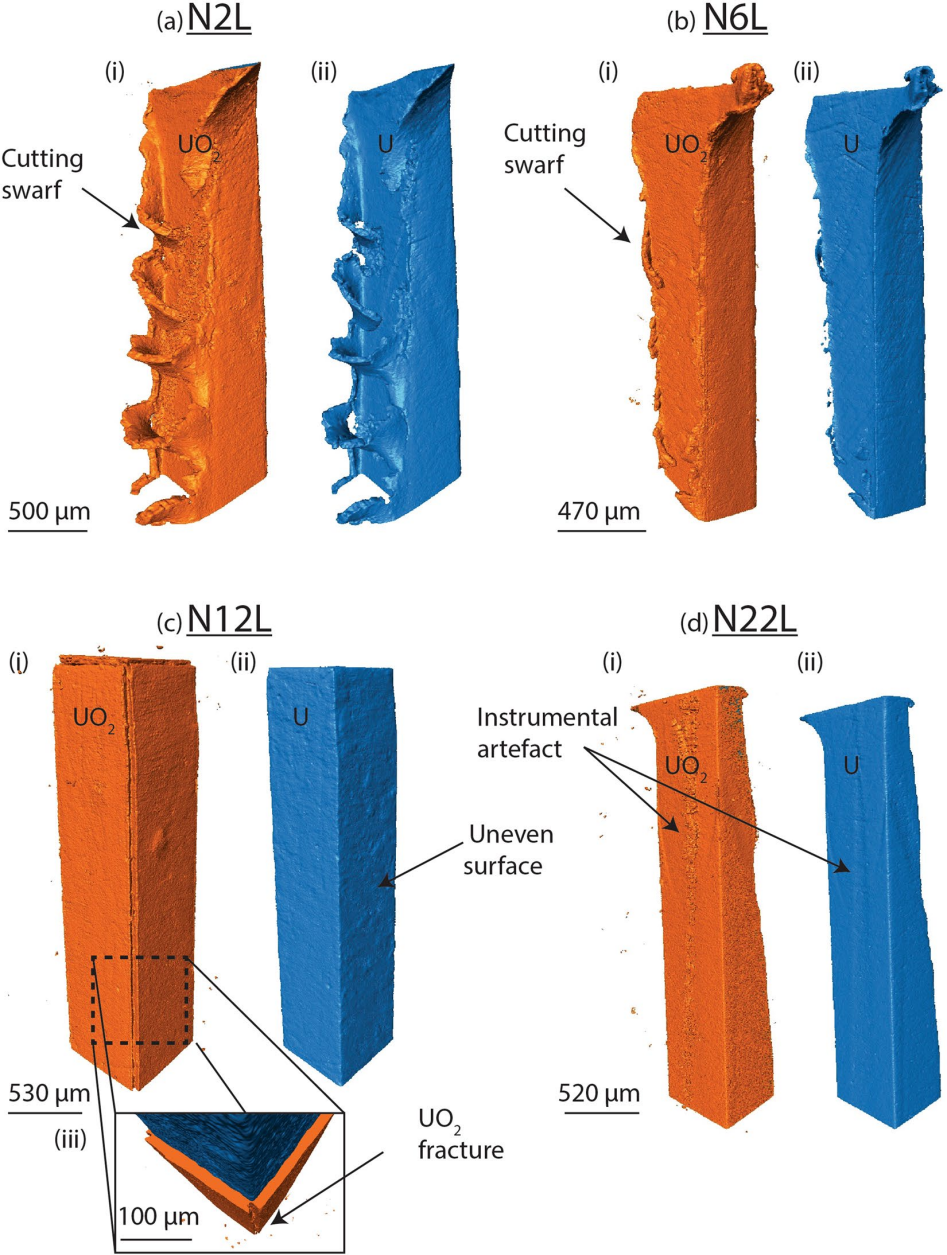
3D renders of the nitric acid etched uranium, encapsulated in grout and submersed in de-ionised water for time periods up to 22 weeks. The corrosion products are exhibited in orange (left) and the uranium metal in blue (right).

XRT data for the ‘as-received’ and nitric acid etched samples are shown in Figs. 2 and 3 respectively.

**Figure 4:**
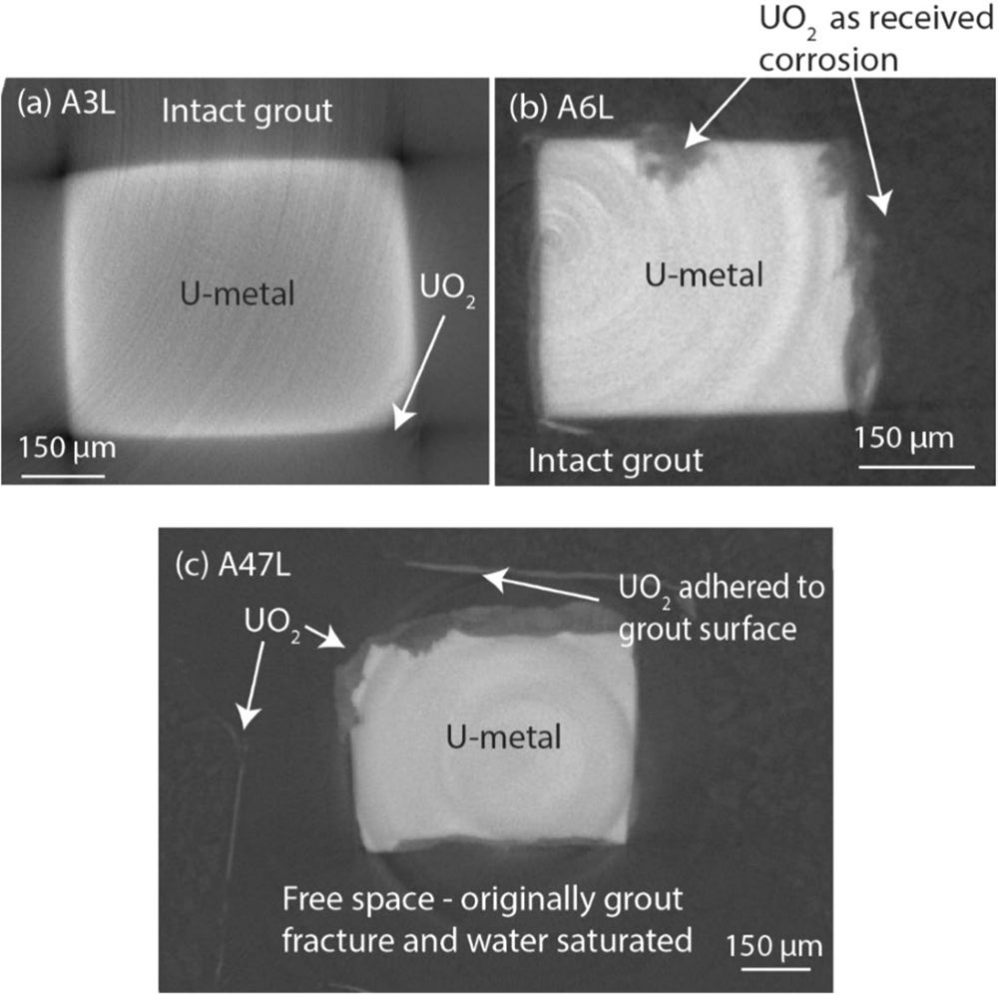
Tomographic cross sections of the as-received samples (A3L-A47L). Note that the cross sections for A3L and A47L are not taken at the same position on the sample.

**Figure 5:**
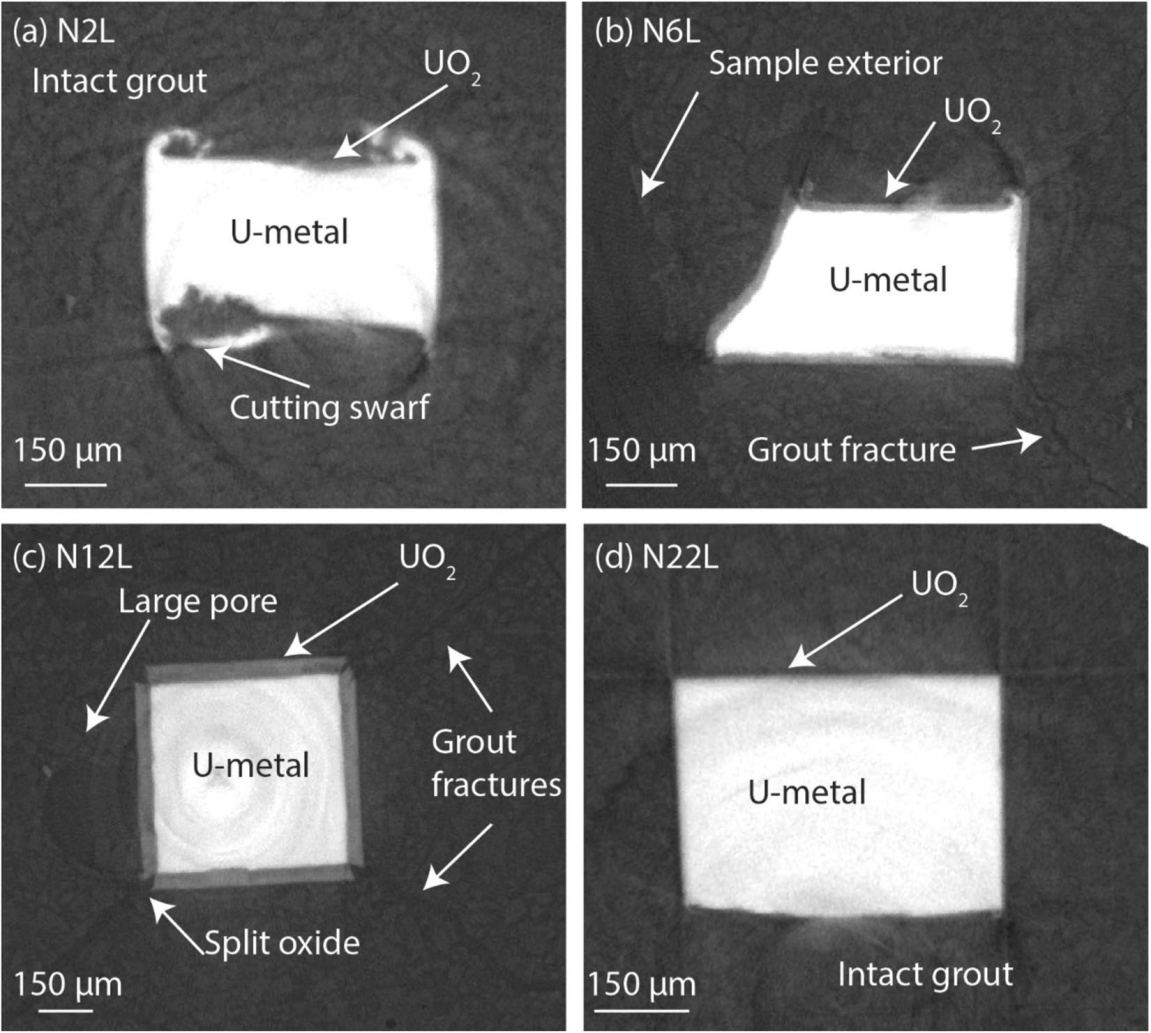
Tomographic cross sections of the nitric acid etched samples N2L-N22L. Note the grout fractures observed in N6L and N12L.

Average oxide thickness measurements were obtained from cross sections of the XRT renders at 80 different positions along the length of each sample and are displayed in Table 2. Selected cross sections of the as-received and nitric acid etched uranium samples are displayed in Figs 4 and 5 respectively.

**Table 2:** The UO_2_ thickness observed on each uranium sample.

Sample	Oxide thickness (µm)	Error + /−(µm)	Range (µm)
A3L	6.68	0.90	7.53
A6L	8.30	0.63	15.21
A47L	31.20	0.50	84.64
N2L	5.42	0.50	10.26
N6L	16.44	0.50	38.15
N12L	39.06	0.50	18.92
N22L	5.25	0.50	11.31

Measurements were obtained from cross sections of the XRT 3D renders. Please refer to Table 2 in^2^ for an explanation on how errors were obtained.

### As-received samples (Group A)

Sample A3L showed an irregular surface, but no significant difference between the uranium metal (Fig. 2(a)(i)) and corrosion product morphology (Fig. 2(a)(ii)) was observed, indicating that if any corrosion had occurred, it was relatively uniform across the metal surface. In comparison, A6L showed an irregular surface which exhibited a degree of pitting (≤250 µm diameter), filled with a corrosion product of ≤40 µm thick. Similar features were observed in characterisation of the metal surface prior to grout encapsulation using secondary electron microscopy imaging shown in the online supplementary (Fig. S1) of our previous study in C. Stitt *et al*.^2^. In agreement with XRPD here, previous Secondary Ion Mass Spectrometry (SIMS) (Supplementary Fig. S2 of ^2^) also detected UO_2_ in addition to carbon, therefore these morphological features are most likely attributed to UO_2_ formation during previous localised water corrosion. Between corrosion masses, the formation of a uniform corrosion layer indicated typical UO_2_ development.

Sample A47L appeared considerably more corroded than when previously analysed as A3L (Fig. 2(c)). Before synchrotron examination, a fracture in the grout was noticed (Fig. 2(iv)) and subsequently during transfer between the reaction and transportation cell, the entire sample disintegrated into three sections (Fig. 2(v)). Inspection of the metal and corrosion products revealed a fine black powder adhered both to the uranium metal and grout where the uranium metal was originally positioned. The sample was reconstructed with Kapton tape before synchrotron examination, permitting analysis of the grout fracture positioning and identification of the corrosion product adhered to the grout and uranium metal. According to XRT measurements, the oxide thickness on sample A47L had increased 5-fold since A3L analysis (Table 2). A reaction front was clearly observed on the uranium metal in Fig. 2(c)(ii), where in this instance, the face of the cuboid had corroded significantly faster than the apex. However, Fig. 4(c) displays greater corrosion around the top left apex and an irregular thickness of oxide around the perimeter of the uranium. This was attributed to material loss during sample transfer and physical reconstruction. Oxide adhered to the grout surface (~10 µm) was not included in the total oxide thickness displayed in Table 2. In addition to oxide formation, small pits correlating with a thicker corrosion product were also exhibited on the A47L uranium metal surface (Fig. 2(c)(iii)). The pits were ~84 µm in diameter with a total corrosion product thickness averaging at 33 µm. These features were not present in the earlier examination of sample A3L, but this may be ascribed to the lower resolution of XRT. However, if not, these features indicate possible UH_3_ formation which were small and scarce enough to prevent XRPD detection. Further investigations are required to ascertain this.

### Nitric acid etched samples (Group N)

The nitric acid etched samples also retained some as-received metal features (Fig. 3). For example, high speed cutting caused excess swarf to form along the cut edges of samples N2L and N6L. On these features, oxide growth was observed to be thicker compared to the flat sample surface. However, overall the nitric acid etched samples showed evidence of typical, relatively uniform oxide growth across the metal surface. Table 2 shows an increase in oxide thickness over time, excluding samples N6L and N12L, which showed noticeably larger average oxide layer thicknesses compared to the remaining samples. Cross sections displaying both the grout and uranium in Figs. 5(b) and(c), reveal that the oxide layer had grown uniformly across all uranium metal faces, excluding the apexes where 17–42 µm wide micro-fractures had formed in the grout and split the oxide at these positions. This morphology was expected of sample A47L, prior to it disintegrating. The metal surface of sample N12L (Fig. 3(c)(ii)) also showed a slightly irregular surface, indicating an uneven distribution of oxide rates across the metal surface. Despite reaction in water for a longer period of time, the oxide thickness exhibited on sample N22L was comparable to that of N2L.

**Figure 6:**
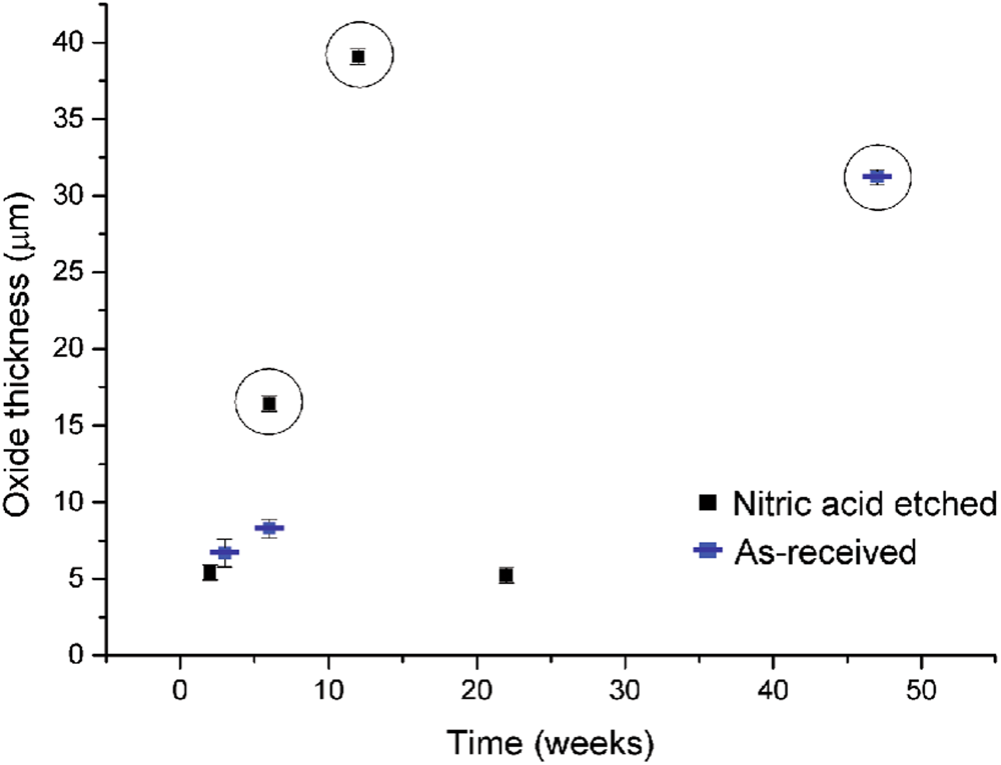
A plot demonstrating the change in UO_2_ thickness on all samples, over time. Circled samples presented grout fracturing. Values and errors are extracted from Table 2.

Figure 6 compares the oxide growth of all the uranium samples encapsulated in grout and exposed to water over different lengths of time. Two oxide growth behaviours were observed: (1) Samples N2L and N22L exhibited similar oxidation thicknesses to the water vapour exposed samples described in^2^. (2) Samples N6L, N22L and A47L showed accelerated corrosion and also displayed grout fracturing. Thus, the occurrence of grout failure appeared independent of the time allowed for corrosion.

### Aqueous chemistry

The pH, Eh and DO content measured from the residual water of each sample were investigated and discussed online in the supplementary material in Figs S1–S3 respectively. Overall, the aqueous conditions exhibited high pH (~pH 12.3), low Eh (~−97 mV) and low dissolved oxygen (DO) (~6.00 mg.L^−1^).

**Figure 7:**
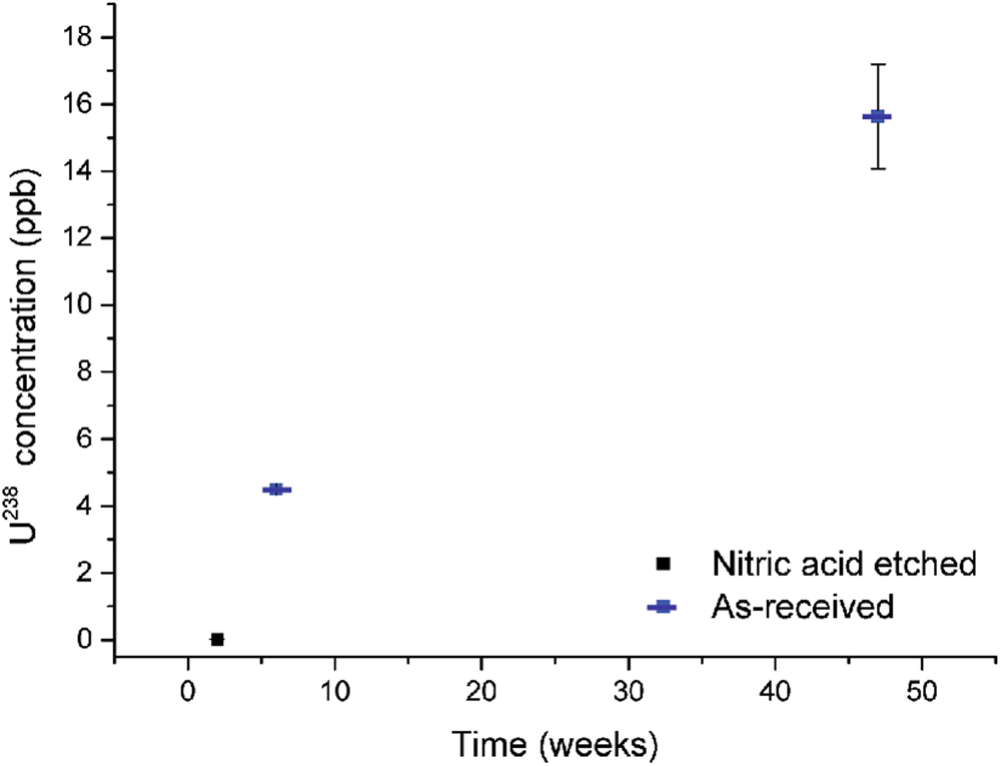
The concentration of ^238^U detected in the residual water of samples N2L, A6L and A42L. For comparison, the average drinking water concentration of uranium in the US in the 1980s was 2.55 ppb^39^.

The concentration of ^238^U in the residual waters of samples N2L, A6L and A47L are displayed in Fig. 7. Over a 47-week period, the concentration of ^238^U increased by an order of magnitude, demonstrating increased mobilisation of uranium within the grout. However, as previously mentioned sample A47L exhibited grout fracturing which may have enhanced uranium mobilisation by directly exposing the metal and UO_2_ to water.

## Discussion

We examined the corrosion behaviour of uranium encapsulated in grout after full submersion in de-ionised water. Each sample was analysed separately using synchrotron XRT and XRPD to identify and determine time dependent development of corrosion product morphology and dimensions. Overall, UO_2_ was considered the dominant corrosion product forming on both the nitric acid etched and as-received uranium metal. It was evident that UO_2_ growth increased over time, with some samples exhibiting accelerated corrosion in comparison to others. Excluding sample A47L, no evidence of uranium hydride formation was present over the 47-week period. Further analysis of sample A47L is required to identify the cause of small localised growths, which may either be indicative of uranium hydride or unidentified as-received corrosion.

**Figure 8:**
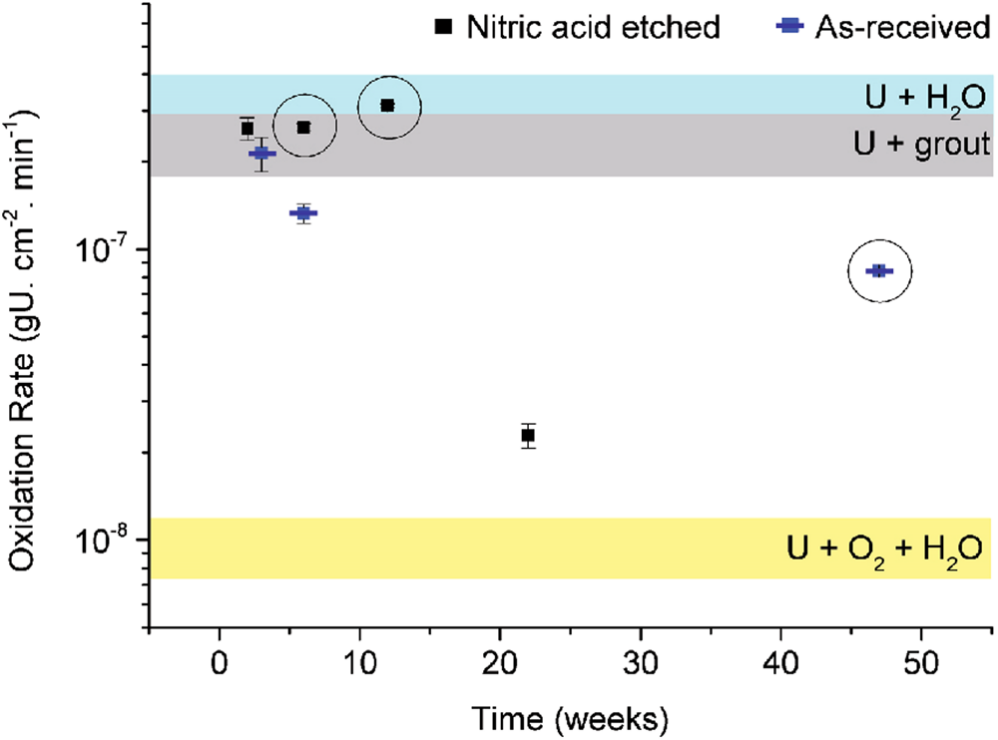
A plot demonstrating the variability in oxide growth rates of grout encapsulated uranium metal, when submersed in water for extended periods of time. Each band represents the oxidation rate at 25 °C, calculated from the empirically derived equations (2–5) displayed at the beginning of this section and sourced from^3,17,18,21,22^. These results are compared to the rates calculated from the oxide thicknesses determined here. The error bars originate from the errors viewed in Table 2.

Oxidation of uranium at room temperature can initially be described as parabolic, as an increasingly thick oxide layer forms on the metal surface. Once the oxide has reached approximately 20–30 nm in thickness, an increased diffusion pathway for oxygen through the existing oxide layer limits formation and ‘old’ oxide on the external surface begins to spall, resulting in a linear oxidation rate^16–20^. A number of linear Arrhenius equations for oxidation rates under varying corrosion conditions have been determined in the literature^3,18,21,22^. To indicate the type of corrosion mechanism occurring in water submersed, grouted uranium metal, the oxidation rates derived from the samples here can be compared to the rates expected using these empirically derived equations (Eqns. 2–5). This is performed in Fig. 8. The average oxide thickness and corrosion time period was used to calculate the rate of oxidation for each sample. For simplicity, the oxidation rate across each sample surface was assumed equal and since the samples were allowed 2 hrs of oxidation in air prior to grout encapsulation, the oxidation rate was assumed to have reached linear rate kinetics^23^. The following Arrhenius expressions were used for comparison:

The U + O_2_ reaction for ≤200 °C from Haschke^21^

2 $$k={e}^{6.19-(\frac{8077}{T})}$$

The U + H_2_O + O_2_ reaction for 25–100 °C from Ritchie^18^ and Delegard and Schmidt^17^

3 $$k=\frac{{10}^{(9.466-(\frac{3836}{T}))}}{60000}$$

The U + H_2_O reaction for 10–350 °C from Delegard and Schmidt^17^

4 $$k=\frac{{10}^{(9.9752-(\frac{3564.3}{T}))}}{60000}$$

The corrosion rates of uranium in BFS/OPC grout determined by Godfrey *et al*.,^3,22^

5 $$k=\frac{3.32\times {10}^{11}\times {e}^{(-\frac{77800}{RT})}}{60000}$$

where the rate *k* = gU.cm^−2^. min^−1^ and temperature T = 299.15–304.15 K and R = 8.314 J.mol^−1^ K^−1^, the universal gas constant.

The oxidation rates of uranium encapsulated in grout and submersed in water showed an overall decrease over time (Fig. 8). Similar to the water vapour exposed samples in reference^2^, the rates commenced with U + H_2_O dominated oxidation for the first 6 weeks, which slowly decreased toward a U + O_2_ + H_2_O oxidation rate with time. Since the aqueous chemistry results suggested low dissolved oxygen and a chemically reducing environment in all samples, this pattern is not attributed to the introduction of oxygen to the system. Instead this was more likely caused by the formation of C-S-H phases (where C = CaO, S = SiO_2_ and H = H_2_O) and mineralisation around grout grains during the first few months of hydration, reducing grout permeability and therefore preventing water diffusion to the metal surface^1,24,25^. The three samples which exhibited grout fracturing (circled in Fig. 8) conveniently demonstrate this point. These samples displayed significantly higher oxidation rates in comparison to samples with intact grout, since their grout fractures provided a pathway for water to access the uranium metal. This behaviour clearly shows that in intact grout, the supply of oxidising species to the uranium metal is a limiting factor to uranium oxidation.

The grout split at all four apexes of the cuboid shaped uranium metal (Fig. 5(c)). Therefore the grout failure observed in samples N6L, N12L and A47L was most likely caused by the volume expansion associated with transformation of uranium to UO_2_ (approximately double). Considering grout fracturing did not occur on sample N22L which had corroded for a longer time period than both N6L and N12L, it is expected an additional source of oxidising species was available for the latter two samples which allowed accelerated development of oxide to thicknesses sufficient to weaken the encasing grout. Establishment of the oxidising species source may have originated from two factors: (1) experimental error and (2) chance. Experimental errors include poor formation of the grout. For example, drying out of the grout during synchrotron preparation (evacuation to 5 × 10^−4^ mbar) at 3 weeks and then re-submersing sample A47L may have weakened the grout by removing essential water required for grout hydration thereby decreasing the grout strength. However, this was not observed to be a problem in the water vapour exposed samples in^2^. Alternatively, the position of the uranium metal within the grout may have been advantageous for the diffusion of oxidising species to the metal surface. The uranium rod in sample N6L was highly angled within the grout and on closer inspection a large portion of the uranium was positioned near the edge of the grout. Figure 5(b) shows a clear breach in the grout between the uranium and exterior water where a water pathway had established.

Sample N12L did not appear to show any grout discrepancies or advantageous positioning of the uranium. However, a large 250 µm diameter pore (Fig. 5(c)) was observed close to the uranium rod, which may have temporarily stored water to fuel uranium oxidation before the grout fractured and subsequently increased water accessibility and further oxidation. If this is the case, a mode of water transport across the metal surface must have been available since the UO_2_ thickness was relatively uniform across all metal surfaces of sample N12L. UO_2_ formed during water oxidation usually yields stoichiometric UO_2.00_, which is reportedly tightly packed and least favourable for diffusion than other oxides^26,27^. However, here the oxide would have probably been non protective, since growth beyond 0.1 µm thickness produces internal stress that cause the oxide to fracture and spall, promoting diffusion through the oxide layer^20^. Consistent with our previous studies^28^, evidence of strong UO_2_-grout bonding was exhibited in sample A47L, but again the XRPD resolution was insufficient to identify any mineralisation at this interface.

The results presented here are consistent with similar spent fuel dissolution experiments^29^; dissolution of ^238^U was slow but nevertheless significant over long periods of time. However, thermodynamically, a high pH, low Eh and low DO environment was expected to discourage the release of uranium metal in water^29,30^. This highlights that the uranium solubility was not necessarily governed by environmental thermodynamics, but instead the transient ion chemistry of the pore water through complexation with aqueous ions^31^. No other elements were investigated through ICP-MS, however given the grout chemistry, the dominating aqueous species were expected to have been the alkali ions NaOH, KOH, Ca(OH)_2_ and uranium in the UO_2_(OH)_3_^−^ form.

To predict the oxide thickness required to cause grout failure, the total oxide thickness believed to be present on the uranium prior to grout encapsulation was calculated. A sample of the same dimensions that had undergone the same preparation as the nitric acid etched samples was allowed to oxidise for 2 hrs in air and was then immediately transferred to SIMS. The oxide thickness was measured at multiple locations using a depth profiling function of UO^+^ (254 amu) and UO_2_^+^ (270 amu) in positive ion mode (30 kV and 3 nA). The average oxide thickness was calculated as 0.076 µm. The maximum oxide thickness observed on an intact, grouted, nitric acid etched uranium sample was 5.42 µm (sample N2L) and the minimum growth on a sample which exhibited grout failure was 15.68 µm (sample N6L), indicating potentially 5.43 µm growth. From these figures and assuming uniform oxide growth across all metal surfaces of a 500 × 500 × 500 µm cube, the range of approximate volume expansions where grout failure could occur without steel confinement, was calculated as 6–19%. Studies of OPC curing under various conditions demonstrated that curing of the grout under water saturated conditions resulted in the grout retaining greater compressive strength and degree of hydration^32–34^, thus these volume expansions are considered conservative for the water vapour exposed samples in^2^.

The oxidation rate of both water vapour exposed^2^ and water submersed (Fig. 8) encapsulated uranium showed evidence of eventually plateauing and reaching a steady state. If the rate of oxidation observed by the water vapour exposed uranium sample A50 (50 week water vapour exposure leading to 7.16 µm average oxide thickness)^2^ continued, then an 19% volume expansion would occur after 2 years of encapsulation. If the oxidation rate continued to decrease toward the U + O_2_ + H_2_O^17^ or the U + O_2_^21^ empirically derived oxidation rates at 25 °C (Fig. 8), then much longer time periods of approximately 4 and 30 years would be required to cause grout failure by uranium oxidation when in unconfined, surface storage or GDF conditions. Considering the Magnox Encapsulation Plant at Sellafield commenced waste encapsulation in 1990, these figures suggest it is likely that some grout fracturing may have occurred within some containers as a result of uranium oxidation^35^. However, these figures neglect the reinforcement provided by the outer stainless steel container and higher compressive stresses present in large volumes of grout.

## Conclusions

The following study examined the *in-situ* corrosion of uranium encapsulated in grout and submersed in de-ionised water. Uranium oxide was identified the dominant corrosion product forming in these conditions and its rate of formation decreased over time. This behaviour was attributed to the decreasing permeability of the surrounding grout and thus reduced access of oxidising species to the metal surface. This theory was further proved as samples containing grout fractures, thus allowing free access for oxidising species to react with the metal surface, showed accelerated oxidation which formed at rates similar to those derived empirically in the literature for the U + 2H_2_O → UO_2_ + 4H_2_ reaction. This correlated well with aqueous chemistry measurements which indicated a chemically reducing and low oxygen environment within the grout, promoting formation of hydrogen. Estimated values were calculated for the volume expansion required to cause grout fracturing in unconfined conditions and these were used to tentatively predict the length of time expected for uranium to cause grout fracturing in waste environments.

## Materials and Methods

### Sample preparation

Both groups (A and N) of 0.5 × 0.5 × 20 mm uranium metal samples underwent exactly the same reaction procedure, bar two differences: (1) the uranium metal source and (2) the uranium preparation procedure. All uranium metal was sourced from Magnox Ltd., however the group labelled A in Table 1 originated from uranium manufactured earlier in the Magnox program than the group labelled N. Over time, increasing concentrations of impurities were added to Magnox uranium metal to improve its reactor properties, however this was not observed to effect the bulk corrosion behaviour. Furthermore, prior to grout encapsulation, Group A did not undergo any uranium surface pre-treatment.

After cutting from a square uranium coupon using a Struers Accutom, the samples in Group A were rinsed and cleaned in water, followed by 5 minutes in a sonicator with acetone and then high purity methanol. Each sample was then left to oxidise in air for two hours to ensure the cut surfaces had reached the linear rate stage of oxide growth. Therefore, at the time of grout encapsulation, Group A retained an ‘as-received’ corrosion layer. The surface features and characterisation are discussed in^2^. These samples were analysed during the first synchrotron session. This excludes sample A47L which was a repeat of sample A3L, 44 weeks after it had been reintroduced to full water submersion immediately after the first beam time.

After cutting from a circular uranium disc, Group N were coarsely abraded from p600–2500 using SiC grit paper and water as lubricant. Each uranium rod was then submersed in 5 M HNO_3_ for 3 hours until they appeared ‘shiny’. These samples were then rinsed and cleaned using the same procedure as Group A and also allowed to oxidise in air for 2 hrs prior to grout encapsulation. Characterisation of the surface after this pre-treatment is also described in^2^.

The metal rods were encapsulated in grout composed of BFS (Redcar Steel Works):OPC (Castle Cement) in a 3:1 ratio and 0.4 w/c. This composition fulfilled the material specification used during ILW encapsulation by Sellafield Ltd., and typical compositions are shown in Utton *et al*.,^36^. Each cylindrical specimen was cured for 3 days in a Perspex mould exposed to a moist environment, before transfer to a reaction cell. The cells composed of a clean glass test tube filled with 17 ml de-ionised water, which was sufficient to completely submerse the grouted uranium sample. Laboratory parafilm was used to seal the top of the test tube. Before synchrotron examination, each sample was transferred to a customised, gas tight, quartz glass-stainless steel reaction cell and evacuated to <5 × 10^−4^ mbar.

### Synchrotron parameters

At the JEEP beam line (Diamond Light Source) XRPD and XRT were performed on each sample separately. For the first beam time energies of 114.6 keV and 115.6 keV were used for XRPD and XRT respectively, however this was reduced to 113.3 keV for both techniques in the second beam time since this energy was further away from the uranium K absorption edge (115.6 keV) and thus produced sharper XRT images. 2D XRPD data were recorded using a flat panel Pixium RF4343 (Thales) in high resolution mode (2880 × 2881 pixels). This detector has a pixel size of 148 × 148 μm. The beam footprint was ~340 × 340 μm on the sample surface. The high resolution PCO pco.4000 imaging detector with its Module 4 camera was used for imaging using a monochromatic beam to obtain the best resolution, 1 pixel = 0.98 × 0.98 μm. Data Analysis WorkbeNch (DAWN) software^37^ was used to view and reconstruct the XRT images of each sample and Avizo® was used to produce 3D renders of the XRT data using the *generate surface* module for specific ranges in greyscale (X-ray intensity) representing each examined material.

Two horizontal XRPD line scans were measured across the 0.5 mm width of each metal sample at two heights along the length of the sample. CeO_2_ was used for beam calibration (NIST - Standard Reference Material 674b). Within the grout, the uranium rod orientation and position changed, resulting in a 1–2 mm variance in sample to detector distance from the central CeO_2_ position. Small shifts in the 2θ value of the XRPD peaks were therefore expected when comparing data. Dawn software for 2D diffraction and processing tools^38^ was used to produce 2D XRPD patterns to 1D.

### Aqueous chemistry

5 ml of the residual water was removed from the N2L, A6L and A47L water to measure the aqueous ^238^U concentration using a VG Thermo Elemental PQ3 ICP-MS. Samples were prepared for ICP-MS by a set dilution in 1% nitric acid (analytical quality concentrated HNO_3_ in Milli-Q water). Blanks and uranium standards were also prepared in 1% nitric acid. An internal bismuth standard of 10 ppb was also added to all blanks, standards and samples.

Data underlying this article can be accessed on Zenodo at https://doi.org/10.5281/zenodo.1118280 and used under the Creative Commons Attribution licence.

## Electronic supplementary material

Supplementary Information
