# Supplementary material for: In-situ, time resolved monitoring of uranium in BFS:OPC grout. Part 2: Corrosion in water
Source: Sci Rep. 2018 Jun 18;8:9282. doi: 10.1038/s41598-018-27636-2 (PMC6006351; doi:10.1038/s41598-018-27636-2)
Supplement: Supplementary file 1 — Supplementary Information [file 41598_2018_27636_MOESM1_ESM.pdf]

In-situ, time resolved monitoring of uranium in BFS:OPC grout. Part 2: Corrosion in water.

C. A. Stitt, C. Paraskevoulakos, A. Banos, N. J. Harker, K. R. Hallam, H. Pullin, A. Davenport, S. Street, T. B. Scott

Aqueous chemistry supplementary information

The aqueous chemistry of the residual water in each reaction cell was investigated immediately after removal of the specimen in an argon filled glove box. The pH, reduction potential (Eh) and dissolved oxygen content (DO) were measured using a Hach HQ40d meter and the PHC201, MTC101 and an optical LDO101 probes respectively. Each probe was left to stabilise in the water for 5 minutes. Additionally,

A standard sample consisting of grout only was also submersed in water for 50 weeks and the Eh and DO measured during the 50th week.
As expected from grout equilibrated water, the pH was high in all sample waters, averaging at pH 12.32 (Fig. S1). The residual water of each sample also exhibited strongly reducing conditions, however over time the Eh became less reducing and begins to plateau near -96.9 mV after 47 weeks (Fig. S2). The recorded Eh value of the plain grout sample was consistent with the uranium containing samples suggesting that OPC:BFS was the primary control on the redox conditions. In agreement with Eh, the DO content was initially low (1.87 $mg.L^{-1}$), but over time this increased to a maximum of ~6.00 $mg.L^{-1}$after 50 weeks (Fig. S3).

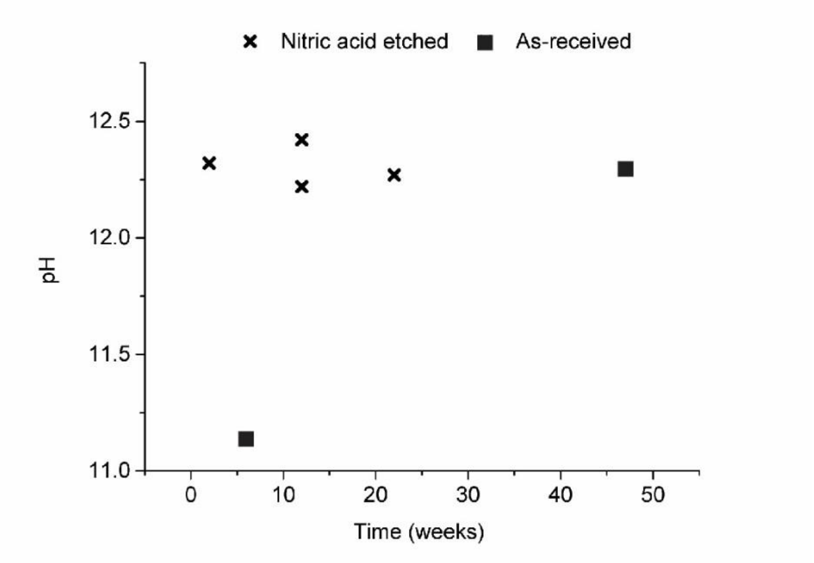

Figure S1. The change in pH measured in the residual water of each sample. An anomaly at 6 weeks displaying pH 11.1 was caused by equilibration with air prior to pH measurement, as this sample was removed from the water earlier than the remaining samples. pH errors ± 0.002

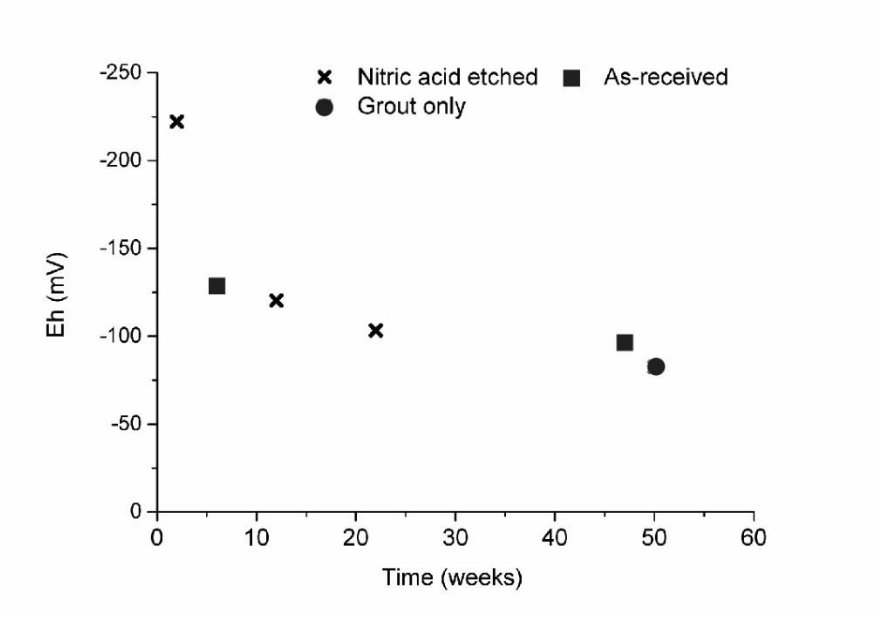

Figure S2. The change in Eh in the residual water of each sample over time. Error ± 0.01 mV.

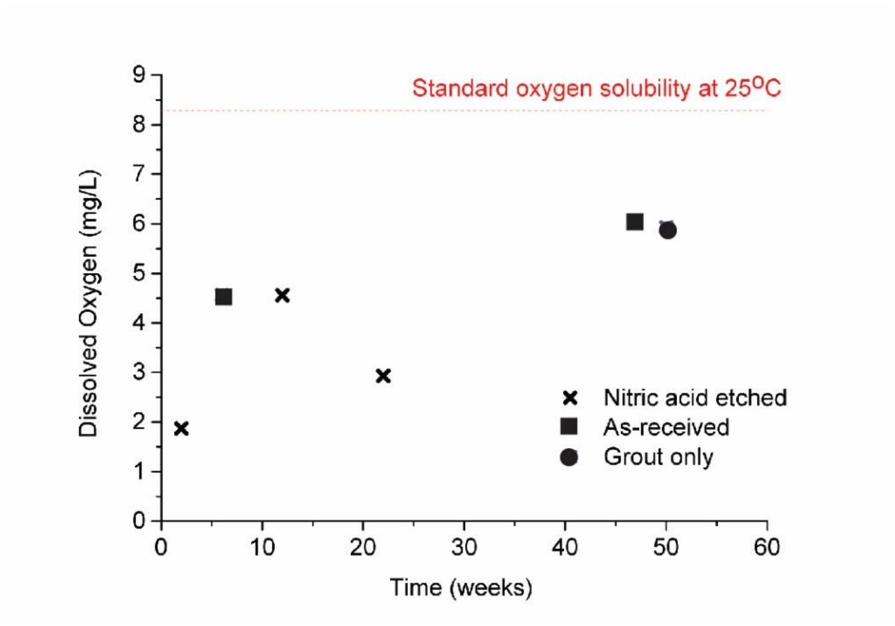

Figure S3. The change in dissolved oxygen levels in the residual water of each sample over time. Errors ± 0.1 $mg.L^{-1}$.
